# Weight censorial score: estimation of the weight loss during concurrent chemo-radiotherapy in nasopharyngeal carcinoma patients by image features predicts prognosis

**DOI:** 10.1007/s11547-025-01953-9

**Published:** 2025-01-28

**Authors:** Jiachen Sun, Sai Kit Edmond Lam, Jiang Zhang, Xinzhi Teng, Francis Kar-ho Lee, Celia Wai-yi Yip, James Chung Hang Chow, Victor Ho Fun Lee, Ying Sun, Jing Cai

**Affiliations:** 1 Department of Health Technology and Informatics, The Hong Kong Polytechnic University, Kowloon, Hong Kong; 2 Department of Clinical Oncology, Queen Elizabeth Hospital, Kowloon, Hong Kong; 3 Department of Clinical Oncology, The University of Hong Kong, Pok Fu Lam, Hong Kong; 4 State Key Laboratory of Oncology in South China, Collaborative Innovation Center for Cancer Medicine, Guangdong Key Laboratory of Nasopharyngeal Carcinoma Diagnostics and Therapy, Department of Radiation Oncology, Sun Yat-Sen University Cancer Center, Guangzhou, China; 5 The Hong Kong Polytechnic University Shenzhen Research Institute, Shenzhen, China; 6 Department of Biomedical Engineering, The Hong Kong Polytechnic University, Kowloon, Hong Kong

**Keywords:** Nasopharyngeal carcinoma, Concurrent chemoradiotherapy, Weight loss prediction, Radiomics, Prognosis

## Abstract

**Purpose:**

Bodyweight loss is commonly found in Nasopharyngeal Carcinoma patients during Concurrent Chemo-radiotherapy (CCRT) and has implications for treatment decisions. However, the prognostic value of this weight loss remains uncertain. We addressed it by proposing a novel index Weight Censorial Score (WCS) that characterizes the patient-specific CCRT response on actual to estimated weight loss.

**Methods:**

A retrospective study included 315 patients from two independent hospitals. An Estimated WCS (eWCS) was obtained through linear regression of image and dosimetry features. The eWCS was converted to an estimated net weight loss (nWL), with its accuracy evaluated. The Determined WCS (dWCS) was calculated by centering and scaling the post-RT actual nWL with patient’s pre-RT body information. The ratio of dWCS to eWCS (WCS ratio) reflected the actual to estimated weight loss of a patient. The prognostic ability of WCS ratio dichotomized at 1 was evaluated.

**Results:**

The mean absolute error of estimated to actual nWL was 1.84 kg. Patients who had their actual WL larger than estimated WL were found to have significantly worse OS (*p* = 0.005, HR = 3.35[1.45–7.73]), PFS (*p* = 0.038, HR = 1.86[1.03–3.35]), and DMFS (*p* = 0.050, HR = 2.20[1.00–4.85]), respectively, in multivariable cox analysis. They were also found not to benefit from adjuvant chemotherapy (*p* = 0.572), whereas the adjuvant chemotherapy provided significant PFS benefit in patients with actual WL smaller than estimated WL (*p* = 0.036, HR = 0.53[0.29–0.96]).

**Conclusion:**

The nWL of patient during CCRT can be reasonably estimated by dosimetry factors at pre-RT stage. The prognostic value of the actual to expected weight loss holds promise for highlighting vulnerable patients after CCRT.

## Introduction

Owing to the radio-sensitive nature of Nasopharyngeal Carcinoma (NPC), high dose radiotherapy (RT) is usually considered as the first line treatment [1]. Nowadays, with more than 70% of NPC cases are detected at an advanced stage, concurrent chemo-radiotherapy (CCRT) has shown superiority than RT alone with respect to progression-free and overall survival in advanced NPC patients and became primary curative treatment choice [2–5]. Considering the anatomical location of NPC are usually juxtaposed to swallowing organs, radiation damage on these functional tissues is unavoidable, resulting significant bodyweight loss (WL) of patients during RT course [2, 6–8]. Concurrent with the additional systemic burden and swallowing dysfunction caused by chemotherapy, there are even higher prevalence and magnitude of WL among patients undergo CCRT than RT alone [9–11].

Percentage weight loss (pWL) of patient after radiation treatment is a common indicator of recently developed malnutrition, which could be intuitively correlate with poor treatment tolerance [12–15]. Despite extensive research had indicated the higher post-RT pWL was significantly associated with worse prognosis, these studies were usually unadjusted, with the prognostic ability of pWL were being outperformed by other common clinical factor, like staging and pre-treatment Body Mass Index (BMI) [16–18]. Another study found contradictory result that the lower pWL during RT was associated with worse prognosis, and suggested the WL without causing malnutrition may produce some beneficial effect of calorie restriction that facilitate tumor control [19]. To date, the prognostic ability and reliability of post-RT pWL is still inconclusive [20].

Medical image analysis in the example of radiomics and dose-volume histogram (DVH) characterize patient’s scan through advanced computational analysis [21]. These pre-treatment image feature offered potentials in predicting RT toxicity before the event happen in NPC patients, examples of oral mucositis, cachexia, and critical weight loss (cWL) [16, 22–24]. Yet, all these studies were found to be binary classification task, and there might be difference in event definition varied across institute’s settings. Several pWL cut-off could be found in defining the cWL threshold for these classification studies [7, 25–28]. Thus, the generalizability of prediction is a concern, and a regression task might be more suitable for analyzing the weight loss issue of NPC patients.

In this study, we aimed to firstly develop a net weight loss (nWL) estimation method by modeling pre-treatment image features, then quantitatively investigate the prognostic ability of this nWL by proposing a novel index Weight Censorial Score (WCS). The WCS ratio characterize each patient’s treatment response by comparing a nWL estimated at pre-CCRT stage to the actual nWL measured at the end of CCRT. We hypothesized that patients would pose worse prognosis if they had their actual nWL greater than the estimated nWL. The purpose of this study was divided into two: 1) assess the accuracy of the nWL estimation at pre-CCRT stage in terms of kilogram (kg) in an external cohort, and 2) validate the ability of the hypothesis-driven WCS ratio for predicting progression-free, overall survival, and adjuvant chemotherapy response.

## Method

### Study sample description

**Fig. 1:**
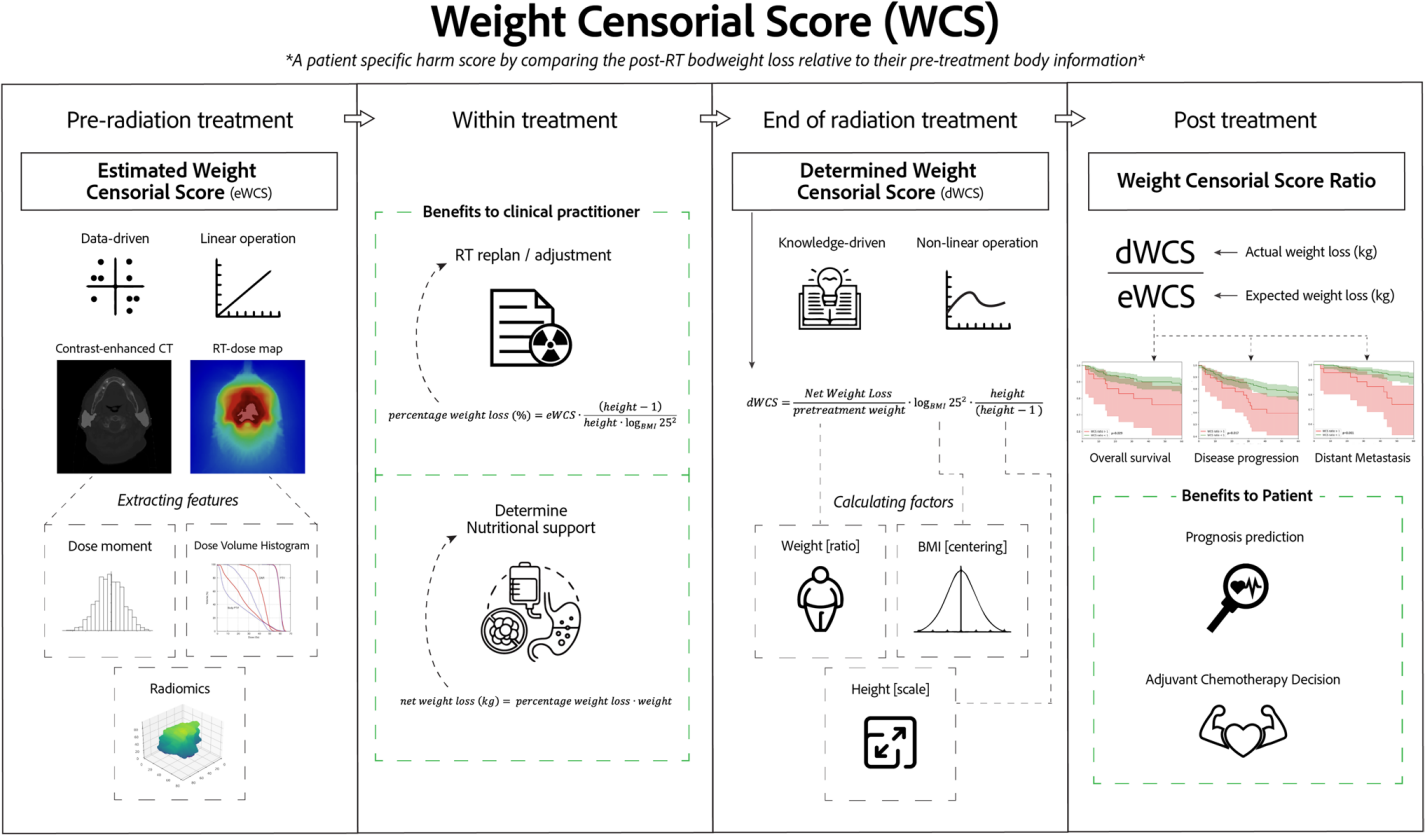
Content diagram of this study. The WCS can be estimated at the pre-RT stage for weight loss prediction, while also can be determined after the RT course for prognostic evaluation

This retrospective study included 699 patients with primary histologically confirmed NPC who underwent concurrent chemo-radiotherapy (CCRT) from 2012 to 2018. The patients had their last follow-up between 2014 and 2021 at Hospital A (*N* = 393) and Hospital B (*N* = 306). Demographic information, including staging, sex, age, histology, treatment arm, pre-treatment BMI, height, and pre-treatment/post-treatment bodyweight was collected. Pre-treatment bodyweight refers to the weight measurement at the beginning of the first RT treatment, while end-treatment bodyweight refers to the weight measurement on the last RT day. The net weight loss (nWL) was calculated by subtracting the end-treatment bodyweight from the pre-treatment bodyweight. Patients who did not experience any weight loss after RT, had insufficient contour information, underwent induction chemotherapy before RT, and with missing end-treatment bodyweight were excluded. A total of 337 patients were included in the study. The training cohort consisted of patients from Hospital A (*N* = 252), while the testing cohort consisted of patients from Hospital B (N = 85). A combined cohort of patients from both hospitals (*N* = 315) was formed for prognosis evaluation after filtering patient’s survival information. A description of the study cohorts, image acquisition, and treatment protocol can be found in Appendix A1-3. A content diagram of this study is shown in Fig. 1.

### Determined weight censorial score definition and justification

The percentage weight loss (pWL) is calculated by comparing the net weight loss (nWL) to the patient's pre-treatment bodyweight (preBW), as shown in Eq. (1). This provides a clear understanding of the impact of weight loss relative to the initial weight, without using specific units.

1 $${\text{pWL}} = \frac{{{\text{nWL}}}}{{{\text{preBW}}}}$$

BMI indicates body fatness, ranging from underweight to overweight. When considering the effect of pWL on individuals with different BMIs, underweight individuals are more susceptible to weight loss compared to obese individuals. To address this, a reference BMI of 25 is chosen. pWL is amplified for patients with a BMI below 25 and reduced for those with a BMI above 25, using a logarithmic transformation with 25 as the base. Squaring the difference further emphasizes the scaling disparity. This adjustment compensates for the greater impact of weight loss on underweight patients when compared to obese patients with the same pWL. The adjusted pWL was shown in Eq. (2).

2 $${\text{adjusted}}\;{\text{pWL}} = ({\text{log}}_{{{\text{BMI}}}} 25)^{2} \times {\text{pWL}}$$

Finally, the determined Weight Censorial Score (dWCS) is calculated by scaling the centered pWL negatively according to the patient’s body height (unit of meter [m]), shown in Eq. (3). Significant weight loss during RT is likely due to daily energy intake being less than energy consumption over time. Taller individuals generally have a higher energy consumption due to their larger body size. While the exact daily energy absorbed is difficult to measure, reduced energy intake through food is observed as the main factor contributing to weight loss in RT patients. If two individuals have the same centered pWL but differ in height, the taller individual will experience a greater net energy gap between absorption and consumption, resulting in more significant weight loss.

3 $${\text{dWCS}} = \frac{{{\text{adjusted}}\;{\text{pWL}} \times {\text{ height}}}}{{\left( {{\text{height}} - 1} \right)}}$$

Equation (3) describes how the dWCS is calculated by centering the pWL based on BMI and scaling it according to height, with the only variable is the nWL measured at the end of RT. The dWCS serves as a harm score that reflects the impact of the nWL observed in patients after RT, censored with their pre-treatment body information. The following regression task of estimated Weight Censorial Score (eWCS) focused solely on the dWCS as the target endpoint.

### Pre-processing and feature extraction

Two image modalities of contrast-enhanced computed tomographic image (CECT) and RT dose stimulated map (dose map) were used for feature extraction. Four targeted regions of interest (ROI) were identified: gross tumoral volume (GTVnp), gross nodal volume (GTVn), combined left and right parotid glands (Parotids), and Larynx. CECT images were resampled to a voxel size of 1 × 1 × 1 mm with a fixed bin width of 25 HU. The dose map was also resampled to 1 × 1 × 1 mm with a fixed bin width of 1 Gy (Gray [Gy]). Image feature extraction was performed separately for each ROI under the two image modalities, including all the features listed in Appendix C1, unless specified otherwise. Shape class features were not extracted on the dose map since they were extracted on CECT. DVH and Moment class features were not extracted for ROIs under CECT. The Parotids feature represents the summation values of the left and right parotid glands. There were 105 features extracted on CECT and 185 features extracted on the dose map for each ROI. DVH and moment features were calculated from our in-house software, and the traditional Radiomics features were extracted through *PyRadiomics* packages.

### Feature selection for estimating weight censorial score

Feature selection was conducted in the training cohort by using a simple statistical test filtering approach, without employing any complex selection algorithm. To determine the significance of the association between each feature and the target variable dWCS, a univariate linear regression test was used. This test computed an F-score with p-value, indicating the significance of the relationship. Features with a p-value greater than 0.05 were removed. In the next step, we calculated the mutual information (cross entropy value) between each significant feature and the target variable. These values were ranked in ascending order. A Pearson correlation test between the significant features was then conducted. If the correlation coefficient (R) between a pair of features exceeded |0.9|, we removed the feature with the lower cross entropy value. All the statistical analyses for feature selection were conducted using the Sci-kit Learn package, with formulas outlined and explained in Appendix B1.

### Modeling estimated weight censorial score

To ensure consistency, the selected features were normalized using min–max scaling, which mapped the values to a range of 0 to 1 based on the data distribution of the training cohort. For modeling, we opted for an ordinary least squares linear regression approach. The objective was to fit a coefficient for each selected feature, minimizing the residual sum of squares between the observed dWCS in the training cohort and the predicted eWCS obtained from the linear approximation. During the model fitting process, no penalty (l1/l2 norm) or cross-validation was applied. The goal was to find the simplest best fit without incorporating any additional constraints or evaluation techniques. To validate the regression performance, samples from the testing cohort were normalized using the scaling parameters obtained from the training cohort. These normalized features were then inputted into the fitted model to generate the eWCS values. All operations were carried out using Sci-kit Learn package with the descriptions provided in Appendix B1.

### Prognostic hypothesis of weight censorial score ratio

4 $${\text{estimated}}\;{\text{nWL}} = \frac{{e{\text{WCS}} \times {\text{preBW}}\; \times \;\left( {{\text{height}} - 1} \right)}}{{\left( {{\text{log}}_{{{\text{BMI}}}} 25} \right)^{2} \; \times \;{\text{height}}}}$$

Since the dWCS is calculated by scaling the end-treatment bodyweight loss with pre-treatment body information, taking the reciprocal step of these calculation allow an estimated nWL (kg) could be obtained from model output eWCS, shown in Eq. 4.

5 $${\text{WCS}}\;{\text{ratio}} = \frac{{{\text{dWCS}}}}{{{\text{eWCS}}}} = \frac{{{\text{actual}}\;{\text{nWL}}\;{\text{measured}}\;{\text{at}}\;{\text{the}}\;{\text{end}}\;{\text{of}}\;{\text{RT}}}}{{{\text{predicted}}\;{\text{nWL}}\;{\text{estimated}}\;{\text{before}}\;{\text{RT}}\;{\text{start}}}}$$

Therefore, the WCS ratio (shown in Eq. 5) provides a comparison between the predicted nWL estimated before CCRT starts and the actual nWL measured on the CCRT-end day. For the WCS ratio of 1 signifies the actual WL is equal to the estimated WL, we chose this value as the cut-off for dichotomizing patients into high- and low-risk groups for prognostic evaluation. Patients with WCS ratio larger than 1 are hypothesized to have a worse prognosis than patients with WCS ratio smaller than 1 as they experienced a higher degree of WL than expected.

### Statistical analysis

The association between dWCS and categorial clinical factors were evaluated by One-way ANOVA test. For the continuous clinical factors, its association with dWCS was assessed by Pearson correlation test. A two-sided *p* < 0.05 was considered significant in both test. The estimated nWL and pWL converted from eWCS were compared with their actual values, respectively, using Concordance Correlation Coefficient (CCC), Coefficient of determination (R^2^), and Pearson correlation test (Metrics explained in Appendix B2), in order to evaluate the prediction agreements. A mean absolute error (MAE) was calculated to assess the accuracy of estimated nWL and pWL at sample-level. Receiver operating characteristics (ROC) curves were generated from the estimated pWL values in predicting cWL, which thresholds were chosen at pWL at ≥ 5%, ≥ 7.5%, and ≥ 10%. The area under the ROC Curve (AUC) was used to evaluate the accuracy of cWL event predictions.

For prognostic evaluation, the starting time of distant metastasis-free survival (DMFS), progression-free survival (PFS), and overall survival (OS) were defined as the end of RT day. The PFS was defined as the time from starting time to either date of disease progression or death from any cause. Patients are dichotomized high- and low-risk groups according to the hypothesized (WCS ratio) value of 1. Survival curves were generated, respectively, for two groups using Kaplan–Meier method and compared by log-rank test. The prognostic value of WCS ratio was evaluated by univariate Cox regression and confirmed under multivariable Cox regression, with hazard ratio (HR) and 95% confidence interval provided. A two-sided *p* < 0.05 was considered significant.

## Results

### Patients’ characteristics

The demographic characteristics of patients in the training cohort and testing cohort, together with their association with dWCS are listed in Table 1. 80% of the patients were suffered from locally advanced stage NPC, with 75.8% of the total patients were male. There were 27.0% (*N* = 68) and 64.8% (*N* = 55) of patients who underwent adjuvant chemotherapy after CCRT in the training and testing cohort, respectively. There was no significant mean difference of dWCS across each subgroup based on tumor stage, nodal stage, sex, treatment arm, NPC histology, and categorized BMI (body fatness) in the training and testing cohort. The dWCS value only showed a significant positive association with the net weight loss (nWL) in the training (*R* = 0.71, *p*-value < 0.001) and testing cohort (*R* = 0.70, *p*-value < 0.001), respectively, but not associated with age, pre-treatment bodyweight, height, and BMI.

**Table 1:** Demographic characteristics, and statistical analysis between the categorical clinical variables and continuous clinical variables with target endpoint determined Weight Censorial Score

Categorical variables	Training cohort (*N* = 252)			Testing cohort (*N* = 85)		
	*N*	Mean	*p*-value	*N*	Mean	*p*-value
*T stage*	0.274			0.510		
1	14	0.267		21	0.218	
2	11	0.215		22	0.243	
3	200	0.240		42	0.244	
4	27	0.272		0	–	
*N stage*	0.778			0.575		
0	0	–		4	0.254	
1	18	0.233		38	0.240	
2	198	0.246		41	0.229	
3	36	0.237		2	0.314	
*Sex*	0.210			0.474		
male	187	0.239		65	0.234	
female	65	0.258		20	0.250	
*Treatment arm*	0.872			0.889		
CCRT*	184	0.245		30	0.239	
CCRT + Adjuvant chemotherapy	68	0.242		55	0.236	
*Histology*	0.762			0.48		
Keratinizing SqCC*	8	0.270		3	0.199	
Differentiated non-keratinizing Carcinoma	9	0.248		1	0.155	
Undifferentiated non-keratinizing Carcinoma	235	0.243		81	0.240	
*Body mass index (BMI)*	0.337			0.396		
< 18.5 (Underweight)	13	0.275		4	0.210	
18.5–22.9 (Slim)	95	0.248		25	0.215	
23–27 (Normal)	106	0.245		35	0.248	
> 27 (Overweight)	37	0.220		21	0.252	

Continuous variables	Pearson's R	*p*-value	Pearson's R	*p*-value
Age	−0.110	0.086	−0.134	0.248
Pre-treatment bodyweight	−0.121	0.055	0.097	0.976
Height	0.072	0.289	−0.090	0.399
Bmi	−0.110	0.087	0.060	0.566
Net weight loss	0.711	** < 0.001**	0.701	** < 0.001**

^*^CCRT Concurrent chemo-radiotherapy, *SqCC* Squamous cell carcinoma

### eWCS development and validation of weight loss prediction

**Fig. 2:**
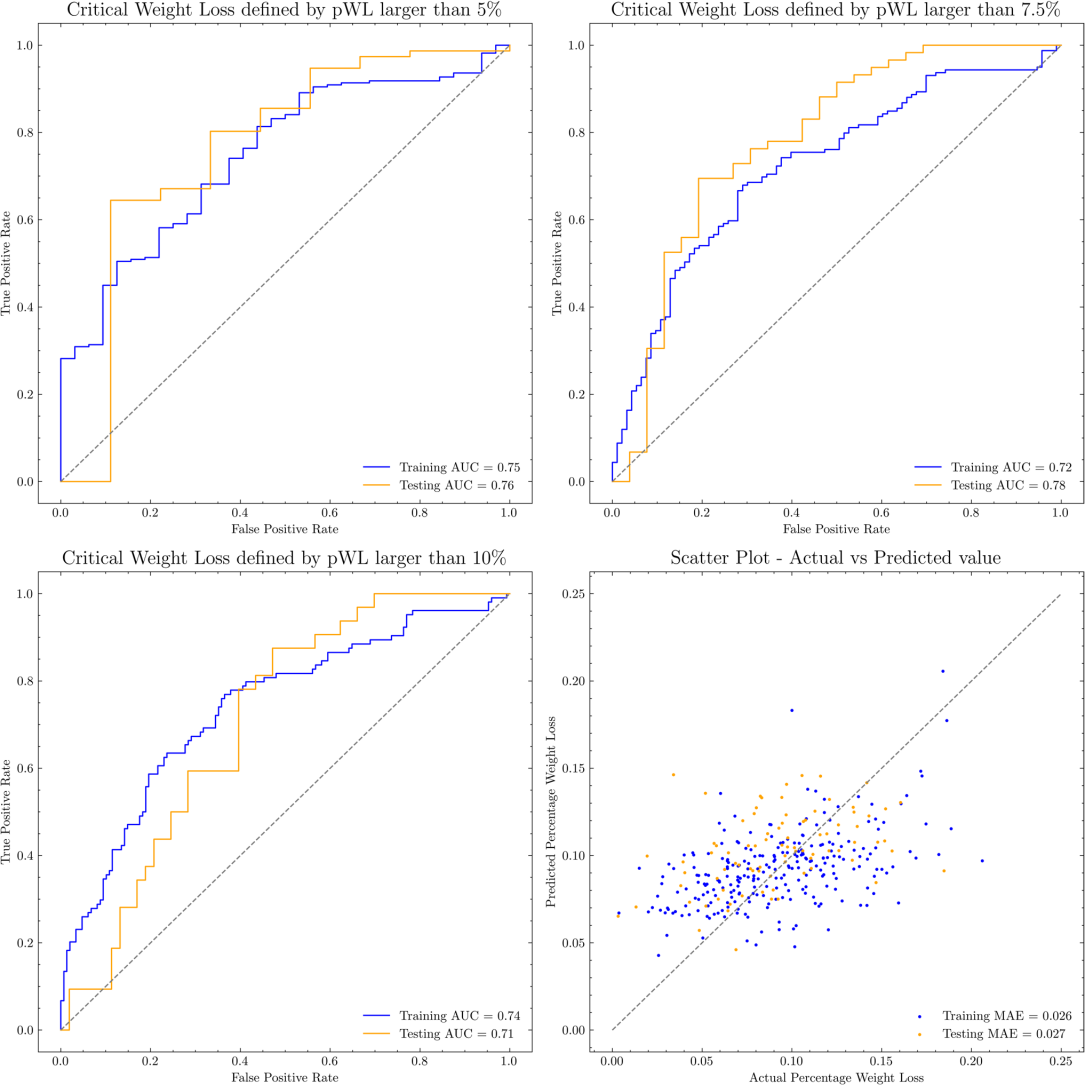
The Receiver Operating Characteristics (ROC) curves of the estimated percentage weight loss in predicting critical weight loss labels, and the scatter plot of estimated to actual percentage weight loss value

20 significant univariate features, with 18 features extracted from the dose map and 2 features extracted from CECT were used to construct the eWCS regression model. The feature equation was provided in Appendix C2. The mean of patient’s actual pWL was 9% in both training and testing cohort. For the training cohort, the mean nWL was 5.99 kg (std = 2.95 kg). For the testing cohort, the mean nWL was 6.33 kg (std = 3.06 kg). A more detailed description of patient’s weight loss data can be found in Appendix A4. Table 2 provided the association and comparison of their estimated values, respectively in the training and testing cohort. The estimated nWL showed the best agreements with its actual values in both cohorts, in terms of CCC (all < 0.642), *R*^2^ coefficient (all < 0.373), and correlation coefficient R (all < 0.680, all *p*-values < 0.001). The mean absolute error (MAE) of estimated to actual nWL was ± 1.694 kg in training cohort and ± 1.838 kg in testing cohort. The MAE of estimated to actual pWL was ± 2.60% in the training and ± 2.70% testing cohort (Table 2B). The ROC curves of estimated pWL in predicting cWL events are shown in Fig. 2. The AUC of the training/testing cohort was 0.75/0.76 for cWL defined at 5%; 0.72/0.78 for cWL defined at 7.5%; and 0.74/0.71 for cWL defined at 10%. A scatter plot of predicted pWL to actual pWL suggesting most predicted pWL was agreed to their actual pWL.

**Table 2:** [A] Association and [B] Comparison between the estimated to the actual values

[A] Association with actual values		Concordance correlation coefficient	Coefficient of determination	Pearson correlation coefficient	*p*-value of Pearson correlation test
Training	eWCS*	0.347	0.210	0.458	** < 0.001**
	predicted pWL*	0.422	0.236	0.492	** < 0.001**
	predicted nWL*	0.668	0.465	0.689	** < 0.001**
Testing	eWCS	0.204	−0.116	0.272	**0.012**
	predicted pWL	0.334	0.014	0.407	** < 0.001**
	predicted nWL	0.642	0.373	0.680	** < 0.001**

[B] Comparison with actual values		Mean absolute error	Mean (std.) value	*p*-value of student T-test
Training	eWCS	0.071	0.244 (0.047)	**1.000**
	predicted pWL	0.026	0.092 (0.021)	**0.824**
	predicted nWL (kg)	1.694	6.090 (2.310)	**0.690**
Testing	eWCS	0.072	0.269 (0.046)	0.005
	predicted pWL	0.027	0.104 (0.022)	0.010
	predicted nWL (kg)	1.838	7.120 (2.530)	**0.069**

^*^eWCS Estimated weight censorial score, *pWL* Percentage weight loss, *nWL* Net weight loss

### Prognostic value of the WCS ratio

**Fig. 3:**
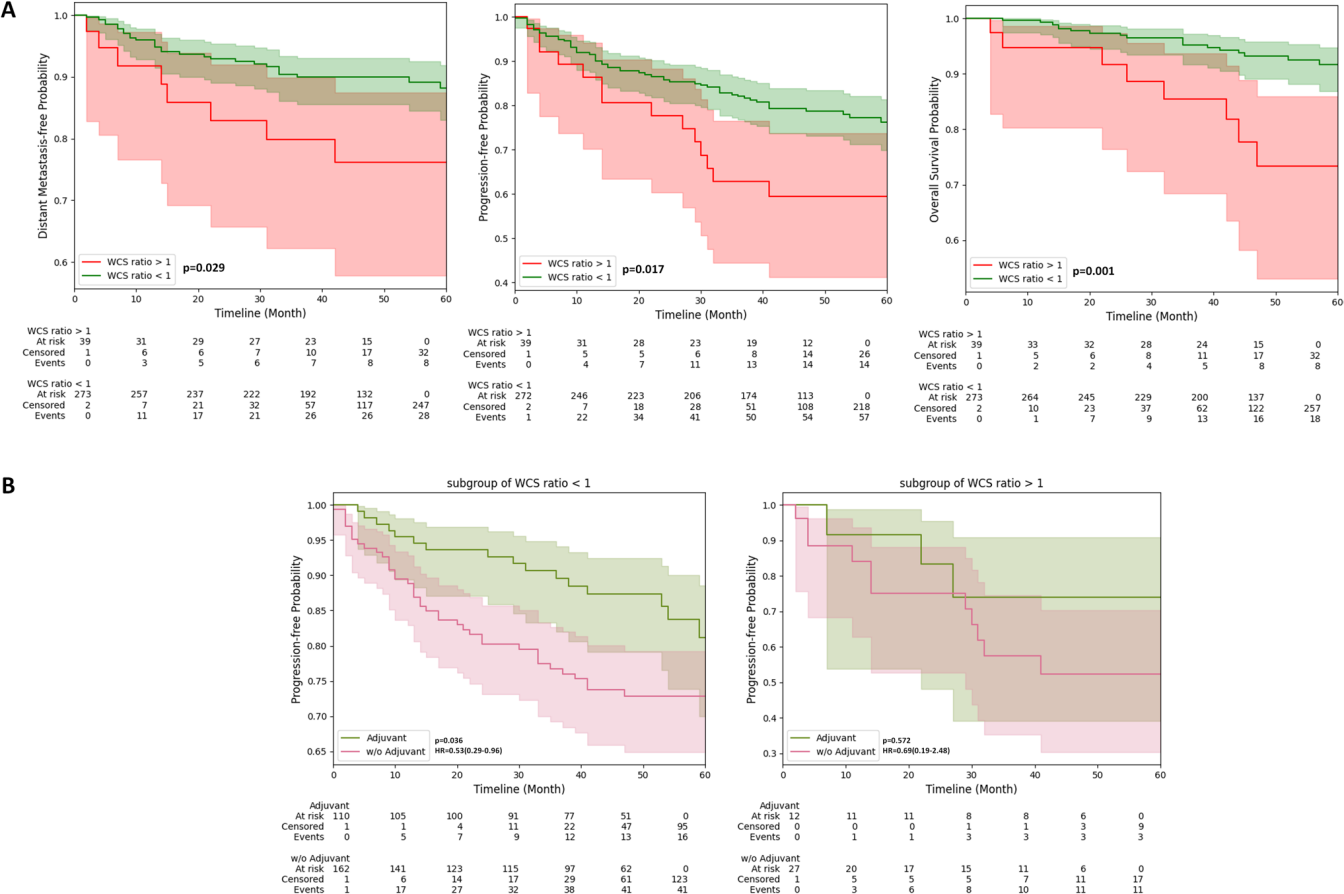
Survival curves of patients dichotomized by Weight Censorial Score ratio and relationship with adjuvant chemotherapy response

The training and testing cohort was combined into one (*N* = 315), with the rate of 5-years OS, PFS, and DMFS rate equaled to 91.74%, 77.46% and 88.25%, respectively (Appendix A4). Patients were dichotomized according to their WCS ratio, with WCS ratio > 1 (actual WL > estimated WL) classified as high-risk group, whereas WCS ratio < 1 (actual WL < estimated WL) classified as low-risk group. For the high-risk group (*N* = 40), the 5-years OS, PFS, and DMFS were 80.00%, 65.00%, and 80.00%, respectively. For the low-risk group (*N* = 275), the 5-years OS, PFS, and DMFS were 93.45%, 79.27%, and 89.82%, respectively. The survival outcomes of the low-risk group were significantly better than the high-risk group (log-rank p-value of OS = 0.001, PFS = 0.017, DMFS = 0.029) as shown in Fig. 3A. The WCS ratio was a significant prognostic factor for OS (*p* = 0.002, HR = 3.66[1.59–8.43]), PFS (*p* = 0.019, HR = 2.02[1.12–3.62]), and DMFS (*p* = 0.034, HR = 2.33[1.06–5.12]) indicated by univariable cox regression (Table 3). Under multivariable cox analysis, the WCS ratio remained an independent factor for predicting OS (*p* = 0.005, HR = 3.35[1.45–7.73]) and PFS (p = 0.038, HR = 1.86[1.03–3.35]), marginally for predicting the DMFS (p = 0.050, HR = 2.20[1.00–4.85]).

**Table 3:** Univariate and Multivariable Cox proportional hazard analysis

Univariate Cox	DMFS		PFS		OS	
	*p*-value	HR (95% CI)	*p*-value	HR (95% CI)	*p*-value	HR (95% CI)
Age > 65 vs. Age < 65	0.257	0.43(0.11–1.82)	0.809	0.91(0.44–1.91)	0.489	1.45(0.50–4.22)
T3/T4 vs. T1/T2	0.218	1.92(0.68–5.44)	*0.022*	*2.64(1.15–6.11)*	0.092	5.58(0.76–41.22)
N2/N3 vs. N0/N1	0.872	0.93(0.39–2.24)	0.441	1.31(0.65–2.65)	0.172	4.02(0.54–29.75)
Male vs. Female	0.210	1.75(0.73–4.21)	0.171	1.51(0.83–2.70)	0.531	0.77(0.33–1.76)
CCRT alone vs. CCRT + AC	0.826	0.93(0.47–1.81)	*0.02*	*1.88(1.10–3.22)*	0.512	1.32(0.57–3.03)
pWL > 10% vs. pWL < 10%	0.066	1.85(0.96–3.58)	0.059	1.56(0.98–2.49)	0.370	1.42(0.66–3.07)
WCS ratio > 1 vs. WCS ratio < 1	*0.034*	*2.33(1.06–5.12)*	*0.019*	*2.02(1.12–3.62)*	*0.002*	*3.66(1.59–8.43)*

Multivariable Cox	*p*-value	HR (95%CI)	*p*-value	HR (95%CI)	*p*-value	HR (95%CI)
T3/T4	0.288	1.76(0.62–5.02)	0.033	2.50(1.04–3.35)	0.119	4.91(0.66–36.47)
WCS ratio > 1	*0.050*	*2.20(1.00–4.85)*	*0.038*	*1.86(1.03–3.35)*	*0.005*	*3.35(1.45–7.73)*

### Predictive value of the WCS ratio for adjuvant chemotherapy response

It was found that for patients in the low-risk group, who had their actual post-RT WL lesser than the pre-RT estimated WL, adjuvant chemotherapy was associated with an improved progression-free survival rate (*p* = 0.036, HR = 0.53[0.29–0.96]). However, in the high-risk group, the adjuvant chemotherapy did not provide significant benefit in terms of progression-free response as shown in Fig. 3B (*p* = 0.572, HR = 0.69[0.19–2.48]).

## Discussion

A significant bodyweight loss during RT is not only common for NPC patients but generally to all head-and-neck cancers (HNC) and lung cancer patients [26, 29–31]. Several studies provided satisfactory results in predicting the post-RT cWL using pre-treatment clinical or image features, with pWL threshold of 4.6%, 5%, 7.5%, and 10% can be found in defining these cWL labels [7, 25–27]. On one hand, these thresholds are often defined by consensus or data-driven approaches that lacking of logical justification for selecting features [32]. On the other hand, the predictive features/model might not be adoptable and generalizable to other institutes due to the difference in clinical definition [25, 33]. To our best knowledge, we performed the first-of-its-kind regression task to estimate the nWL (in kg) of NPC patients during CCRT with pre-treatment information. The regression feature was selected and modeled not solely on the nWL itself, but on weight loss ‘score’ that was scaled and adjusted by extra pre-treatment body information. These mathematical tricks maintained the prediction robustness in a generalized cohort, allowing the value of patient’s weight loss rather than a binary event can be predicted and adopted in different clinical set-ups.

The magnitude of pWL during CCRT of each patient can be varied significantly due to their intrinsic difference in body physique, dietary habits, and willingness [20, 34]. Moreover, the prognostic effect of pWL is susceptibly associated with malnutrition, weaken immunity defense system, and tumor potentiation [11, 19, 35]. Taking the phenomenon (weight loss) of ground truth causes as a prognostic factor is not warrantable, resulting weak or even contradictory prognostic result found in pWL among NPC patients [20]. In contrast, the eWCS provides image characterization on tumor and radiation dosimetry; the dWCS provides patient characterization on actual bodyweight loss and body information. Several aspects that related to radiation and bodyweight were considered into a single index (WCS ratio) for evaluation, resulting significant association could be found with OS, PFS, and DMFS. Further the prognostic result of WCS ratio is more explainable, as the WL was solely estimated from image features, two patients with one experienced higher actual WL must indicate a poorer tolerance of the same treatment condition. Thus, the unidirectional association of WCS ratio and prognosis is granted. We found that the WCS difference, i.e., dWCS – eWCS and dichotomized at 0 was also significantly associated with these survival events (Results demonstrated in Appendix D1-2). The WCS ratio and WCS difference share the same concept and warrant further analysis. While the nWL difference and pWL difference can be converted from dWCS and eWCS, the WCS difference outperformed these indicators in our study cohort (Appendix D3).

Adjuvant chemotherapy (AC) with CCRT are first-line options for treatment of loco-regionally advanced NPC. However, these adjuvant platinum regimens are poorly tolerated, with several multicenter trials reported that only around 60% to 70% of patients could tolerate the entire AC regimen [36]. For post-RT systemic inflammation and malnutrition patients, these vulnerable patients had nearly 4.5-fold increased risk of death in AC as reported from recent multi-cohort study [37]. Most studies had confirmed the efficacy of concurrent chemotherapy during RT, while the role of AC remained undefined but is associated with increased toxicity [37, 38]. In our study, the WCS dichotomization highlighted a subgroup of patients who had their actual WL smaller than their estimated WL after CCRT, and found to be significantly benefit from the AC regimen in terms of disease progression (HR = 0.53[0.29–0.96]). In contrast, around 13% of patients (*N* = 40) in our study cohort had experienced an actual WL higher than the estimated WL. The AC after CCRT did not provide a significant progression benefit on them (*p* = 0.572, HR = 0.69[0.19–2.48]). The idea of WCS ratio could be thought as the patient’s recovery ability compared to the definitive CCRT toxicity, that holds a promising identification of vulnerable patients who might not be suitable for or tolerating further AC treatment. This allows the immediate re-adjustment of treatment plan at the end of CCRT without extra cost.

The study hypothesizing 1 to signify high- and low-risk group among patients in terms of WCS ratio, and found it significantly associated with prognosis. Dichotomize the WCS ratio larger than 1 would increase the hazard ratio accordingly, that behave similar with defining a threshold on pWL. Further studies are suggested to define their own threshold value on WCS ratio for specific evaluation, examples of oral mucositis and sinus mucosa abnormalities, or late complications like osteoradionecrosis and xerostomia [39]. The WCS ratio is just a comparison between the WL reflected on patient’s body to the WL estimated from medical images, that the dWCS can be calculated at any time during CCRT and compare with the fixed eWCS. In this scenario, the WCS ratio might act as indicator of developing complication and necessity of RT-replan. Dichotomizing the WCS ratio smaller than 1 is then worth for consideration. Besides abovementioned, the eWCS also provide significant benefit in pre-treatment stage. Current nutritional factor like post-RT pWL or Karnofsky Performance Score (KPS) could only indicate complications had been developed in patients [40]. While eWCS provides a sign of possible complication (malnutrition) might develop during treatment, vulnerable patient or faulty dosimetry plan could be identified before treatment commence. Thus, early intervention, rehabilitation training, and medication could be offered and prescribed to patients in an earlier stage. We also believed that the prevention of WL during CCRT should be done by both practitioners and patients themselves, informing the estimated WL to patients might allow better mental and physical preparation before the complication occur.

The major limitation of this study is the retrospective nature, that we could only combine the training and testing cohort as one for prognosis evaluation due to small cohort size. Although the prognostic ability of WCS ratio has not been validated, the dichotomization was obtained by logical hypothesis but not driven by data, which provides credibility for validating in an external cohort in the future. Another limitation of this study was the demographics bias. Despite samples were recruited from two independent hospitals, most of the patients were in advanced stage, all treated with CCRT, and all of them were southern Chinese. Firstly, the additional side effects caused by chemotherapy drug were also strongly associated with severe WL, examples of nausea, sore mouth, and loss of appetite. These variables should be considered and adjusted before applying WCS on patient with RT alone regimen. Secondly, the current WCS equation did not consider effects from age, sex, and ethnicity, which pose difference in terms of living and dietary habit that might affect the degree of WL. The formulation of WCS could be further enhanced to broaden its impact. On the other side, the actual impact and significance of estimating nWL/pWL in decision-making cannot be validated here without clinical labels, despite we could accurately estimate the nWL/pWL of a patient at pre-RT stage. The accurate prediction of weight loss does not guarantee successful maintenance through nutritional support, and patients with no weight loss during RT may still experience harm. Future studies are encouraged to analyze the association of eWCS with inflammatory markers and tube feeding labels.

## Conclusion

Pre-RT dosimetry and image features could give an accurate estimation of patient’s weight loss during CCRT. The ratio of actual weight loss to estimated weight loss can be characterized by WCS ratio, which is an interpretable and independent prognostic marker for disease progression. Patients with their actual weight loss smaller than estimated weight loss were found to benefit from adjuvant chemotherapy.

## Data Availability

Research data are stored in an institutional repository and will be shared upon request to the corresponding author.

## Appendix

[A] Description of the study cohort, image acquisition, and treatment protocol page 2–3.

[B] Statistics and metrics for feature selection, modeling, and evaluation page 4–5.

[C] Feature table, eWCS feature equation, and secondary modeling equation page 6–7.

[D] Study summary and additional prognostic evaluations on weight loss page 8.

## Appendix A: Description of the study cohort, image acquisition, and treatment protocol

### [A2] Image acquisitions

The contrast-enhanced CT scan used for RT planning of enrolled patients, where retrospectively retrieved in Digital Imaging and Communication in Medicine (DICOM) format, archived using Picture Archiving and Communication System (PACs). For the image acquisition, patients were immobilized with thermoplastic cast in supine position. The scan range covered from the vertex to 5 cm below the sternoclavicular notch and was acquired at 3 mm intervals using a 16-slice Brilliance Big Bore CT (Philips Medical Systems, Cleveland, OH). With helical scan mode, voltage at 120 kVp, current at 254 mA with 325 ms exposure time. The pixel spacing of acquisition matrix is in 1.152 × 1.152 mm, with size 512 × 512 at 3 mm slice thickness. One of the two types intravenous contrast agents were used considering patient’s allergy history: OMNIPAQUE TM 350 mg I/ml or VISIPAQUE TM 320 mg I/ml, with 70 ml prescribed at a injection rate of 2 ml/sec. Scanning performed after 30 s of time delay. For the RT dose stimulation map, it was designed and acquired from the treatment plans using Pinnacle treatment planning system v 9.0. The grid spacing for displaying the dosimetry was in 1 × 1 × 1 mm.

### [A3] Treatment protocol

Concurrent chemotherapy was conducted every three weeklies with 100 mg/m^2^ cisplatin during the Intensity-modulated Radiotherapy (IMRT). The IMRT practicing slightly different regarding the contouring of high risk (HR) and low risk (LR) clinical target volumes (CTV) of the nasopharynx (NP) and lymph nodes (LN), total tumor dose prescription and fractionation, dose constraints of organs at risk, radiation plan acceptance criteria and beam arrangements. In general, the gross tumor volume (GTV) for nasopharyngeal primary (GTV NP) and cervical lymph nodes (GTV LN) were determined from the imaging, clinical and endoscopic findings. The CTV NP (HR) covers the GTV NP with 5–10 mm margin, the CTV NP (LR) covers the CTV NP (HR) plus areas at risk for microscopic involvement. The CTV LN (HR) covers GTV LN with 5–10 mm margin and CTV LN (LR) covers CTV LN (HR) plus level Ib to V nodal regions of neck contoured according to International Consensus Guidelines for the CT-based delineation of neck levels. The dose to GTV NP and GTV LN were 66–74 Gy and 66–70 Gy in 33–35 fractions, respectively, with or without simultaneous integrated boost (SIB). Additional planned boost to beyond 70 Gy (EQD2) to the nasopharynx or lymph nodes were delivered at the discretion of oncologist to achieve dose escalation. Both CTV NP (HR) and CTV LN (HR) also received 66–70 Gy in 33–35 fractions while the CTV NP (LR) and neck CTV LN (LR) received 60–62 Gy in 33–35 fractions. For adjuvant phase, patients allocated to AC arms [AC–cisplatin-fluorouracil combination (PF)] were given 3 cycles of adjuvant cisplatin (80 mg/m^2^ on day 1) and 5-fluorouracil (5-FU; 1,000 mg/m^2^/day on day 1–4) every 28 days for three cycles.

### [A4] Description of the weight loss events and survival labels in the training, testing, and combined cohort

**Table:**

[A] Weight loss event	Training cohort (*N* = 252)			Testing cohort (*N* = 85)		
	nWL (kg)	pWL	dWCS	nWL (kg)	pWL	dWCS
Mean	5.99	0.09	0.24	6.33	0.09	0.24
Std	2.95	0.04	0.11	3.06	0.04	0.09
Range	0.20–17.25	< 0.01–0.21	0.01–0.54	0.20–13.20	< 0.01–0.18	0.01–0.51

**Table:**

[B] prognostic event	Combined cohort (*N* = 315)					
	OS		PFS		DMFS	
5 years event-free rate	91.74%		77.46%		88.25%	
	Stratified on WCS ratio > 1					
	High-risk group (*N* = 40, 12.70%)			Low-risk group (*N* = 275, 87.30%)		
	OS	PFS	DMFS	OS	PFS	DMFS
5 years event-free rate	80.00%	65.00%	80.00%	93.45%	79.27%	89.82%

	Stratified on WCS ratio > 1					
	High-risk group (*N* = 40, 12.70%)			Low-risk group (*N* = 275, 87.30%)		
	OS	PFS	DMFS	OS	PFS	DMFS
5 years event-free rate	80.00%	65.00%	80.00%	93.45%	79.27%	89.82%

## Appendix B: Statistics and metrics for feature selection, modeling, and evaluation

### [B1] Statistics method for feature selection and modeling

All the statistics analysis for feature selection, feature normalization before modeling, and linear regression are executed with Scikit-learn package, with the API description provided below:

F-score univariate feature selection.

https://scikit-learn.org/stable/modules/generated/sklearn.feature_selection.f_regression.html

Estimating mutual information.

https://scikit-learn.org/stable/modules/generated/sklearn.feature_selection.mutual_info_classif.html

Pearson correlation testing.

https://scikit-learn.org/stable/modules/generated/sklearn.feature_selection.r_regression.html

Min–max normalization.

https://scikit-learn.org/stable/modules/generated/sklearn.preprocessing.MinMaxScaler.html

Ordinary least squares Linear Regression.

https://scikit-learn.org/stable/modules/generated/sklearn.linear_model.LinearRegression.html

### [B2] Metrics for evaluating the estimated net/percentage weight loss and weight censorial score

The Concordance correlation coefficient (CCC) measures the agreement between the pair of predicted and actual variables, considering both precision and accuracy from -1 to 1 with 1 is the best agreement. This is usually to evaluate the reproducibility of an accurate weight loss to be accurately estimated. Computing N paired predict-actual values (${y}_{n},\widehat{{y}_{n}}$) for *n* = 1, 2, …, N, the CCC is defined as:

$$\rho_{c} = \frac{{{\text{Expected}}\;{\text{orthogonal}}\;{\text{squared}}\;{\text{distance}}\;{\text{from }}\;{\text{the }}\;{\text{diagonal}}\;y = \hat{y}}}{{{\text{Expected}}\;{\text{orthogonal}}\;{\text{squared}}\;{\text{distance}}\;{\text{from}}\;{\text{the}}\;{\text{diagonal}}\;y = \hat{y}\;{\text{assuming}}\;{\text{independence}}}}$$

With the mean, variance, covariance, and Pearson correlation coefficient can be calculated by:

$$\overline{y }= \frac{1}{N}\sum_{n=1}^{N}{y}_{n}$$

$${s}_{y}^{2}= \frac{1}{N}\sum_{n=1}^{N}{({y}_{n}- \overline{y })}^{2}$$

$${s}_{y\widehat{y}}= \frac{1}{N}\sum_{n=1}^{N}{(y}_{n}-\overline{y })({\widehat{y}}_{n}- \overline{\widehat{y} })$$

$$\rho =\frac{{s}_{y\widehat{y}}}{{s}_{y}{s}_{\widehat{y}}}$$

The CCC can be calculated by:

$${\rho }_{c}= \frac{2\rho {s}_{y}{s}_{\widehat{y}}}{{s}_{y}^{2}+ {s}_{\widehat{y}}^{2}+{(\overline{y }-\overline{\widehat{y} })}^{2}}$$

The coefficient of determination (${R}^{2}$) is the proportion of the variation in the predictable dependent variable from the independent variables. It provides a measurement on how well does the actual weight loss are replicated by the model’s estimated weight loss, abased on the proportion of total variation of outcomes explained by the regression model. The ${R}^{2}$ is defined as:${R}^{2}=1-\frac{{SS}_{\text{residual}}}{{SS}_{\text{total}}}$

With the sum of squares of residuals, and total sum of squares can be calculated by:

$${SS}_{\text{residual}}= \sum_{n=1}^{N}{({y}_{n}-\widehat{{y}_{n}})}^{2}$$

$${SS}_{\text{total}}= \sum_{n=1}^{N}{({y}_{n}-\overline{y })}^{2}$$

Note that if $\overline{\widehat{y} }$ differs a lot from $\overline{y }$, the prediction does not explain the target variance at all, may resulting a negative ${R}^{2}$.

## Appendix C: Feature table, eWCS feature equation, and secondary modeling equation

### [C1] Feature Table

$${\text{The}}\;{\text{feature }}\;\left\{ {{\text{Class}}} \right\}_{{\left\{ {{\text{Name}}} \right\}}} \;{\text{of}}\;\left\{ {ROI} \right\}\;{\text{extracted}}\;{\text{on}}\;{\text{Image}}\;\left\{ {{\text{Modality}}} \right\} \equiv {\text{Class}}_{{{\text{Name}}ROI}}^{{{\text{Modality}}}}$$

**Table:**

Feature class	Class description	Feature name
Shape (*N* = 14)	2D/3D shape-based description	Elongation, Flatness, Least Axis Length, Minor Axis Length, Major Axis Length, Mesh volume, Maximum 2D Diameter Column, Maximum 2D Diameter Row, Maximum 2D Diameter Slice, Maximum 3D Diameter, Sphericity, Surface Area, Surface Volume Ratio, Voxel Volume
Energy (*N* = 18)	1st order intensity statistics	10Percentile, 90Percentile, Energy, Entropy, Inter-quartile Range, Kurtosis, Maximum, Mean Absolute Deviation, Minimum, Mean, Median, Range, Robust Mean Absolute Deviation, Root Mean Squared, Skewness, Total Energy, Uniformity, Variance
Moment (*N* = 64)	Weighted average of voxels with different order in x, y, z, axis ${\sum }_{x}{\sum }_{y}{\sum }_{z}{x}^{i}{y}^{j}{z}^{k} I(x, y, z)$	001, 002, 003, 010, 011, 012, 013, 020, 021, 022, 023, 030, 031, 032, 033, 100, 101, 102, 103, 110, 111, 112, 113, 120, 122, 123,130, 131, 132, 133, 200, 201, 202, 203, 210, 211, 212, 213, 220, 221, 222, 223, 230, 231, 232, 233, 300, 301, 302, 303, 310, 311, 312, 313, 320, 321, 322, 323, 330, 331, 332, 333
DVH (*N* = 30)	Dose volume histogram-based statistics	D40, D45, D50, D55, D60, D65, D70, D75, D80, D85, D90, V30, V35, V40, V42, V44, V46, V48, V50, V52, V54, V56, V58, V60, V62, V64, V66, V68, V70, V72
GLCM (*N* = 22)	Gray level co-occurrence matrix-based statistics	Autocorrelation, Joint Average, Cluster Prominence, Cluster Shade, Cluster Tendency, Contrast, Correlation, Difference Average, Difference Entropy, Difference Variance, Joint Energy, Joint Entropy, IMC1, IMC2, IDM, IDMN, ID, IDN, Inverse Variance, Maximum Probability, Sum Entropy, Sum of Squares,
GLDM (*N* = 14)	Gray level dependence matrix-based statistics	SDE, LDE, GLN, GLNN, DN, DNN, GLV, DV, LGLE, HGLE, SDLGLE, SDHGLE, LDLGLE, LDHGLE
GLRLM (*N* = 16)	Gray level run length matrix-based statistics	SRE, LRE, GLN, GLNN, RLN, RLNN, RP, GLV, RV, RE, LGLRE, HGLRE, SRLGLE, SRHGLE, LRLGLR, LRHGLE
GLSZM (*N* = 16)	Gray level size zone matrix-based statistics	SAE, LAE, GLN, GLNN, SZN, SZNN, ZP, GLV, ZV, ZE, LGLZE, HGLZE, SALGLE, SAHGLE, LALGLE, LAHGLE
NGTDM (*N* = 5)	Neighboring gray tone difference matrix-based statistics	Coarseness, Contrast, Busyness, Complexity, Strength

The formula and definition of features can be found in the pyradiomics API https://pyradiomics.readthedocs.io/en/latest/features.html

### [C2] The linear regression equation of features for estimated weight censorial score value

**Table:**

Region of interest	Feature name	Feature weight (coefficient)
Gross tumor volume (GTVnp)	${{\text{Energy}}_{90\text{Percentile}}}_{\text{GTV}np}^{\text{CECT}}$	-0.037
	${{\text{Moment}}_{112}}_{\text{GTV}np}^{\text{Dose}}$	0.212
Gross nodal volume (GTVn)	${{\text{Moment}}_{121}}_{\text{GTV}n}^{\text{Dose}}$	0.325
	${{\text{Moment}}_{221}}_{\text{GTV}n}^{\text{Dose}}$	-0.058
	${{\text{Moment}}_{301}}_{\text{GTV}n}^{\text{Dose}}$	-0.043
	${{\text{Moment}}_{303}}_{\text{GTV}n}^{\text{Dose}}$	0.132
	${{\text{Moment}}_{311}}_{\text{GTV}n}^{\text{Dose}}$	0.074
	${{\text{Moment}}_{320}}_{\text{GTV}n}^{\text{Dose}}$	-0.207
	${{\text{Moment}}_{323}}_{\text{GTVn}}^{\text{Dose}}$	-0.184
	${{\text{GLRLM}}_{\text{LRLGLE}}}_{\text{GTV}n}^{\text{Dose}}$	0.080
Combined parotid glands (Parotids)	${{\text{GLRLM}}_{\text{RE}}}_{\text{Parotids}}^{\text{CECT}}$	-0.059
	${{\text{Moment}}_{003}}_{\text{Parotids}}^{\text{Dose}}$	-0.101
	${{\text{Moment}}_{032}}_{\text{Parotids}}^{\text{Dose}}$	0.169
	${{\text{GLSZM}}_{\text{LGLZE}}}_{\text{Parotids}}^{\text{Dose}}$	0.108
Larynx	${{\text{Energy}}_{\text{Minimum}}}_{\text{Larynx}}^{\text{Dose}}$	-0.090
	${{\text{Energy}}_{\text{Range}}}_{\text{Larynx}}^{\text{Dose}}$	-0.038
	${{\text{Energy}}_{\text{Uniformity}}}_{\text{Larynx}}^{\text{Dose}}$	0.011
	${{\text{GLCM}}_{\text{Correlation}}}_{\text{Larynx}}^{\text{Dose}}$	-0.086
	${{\text{GLSZM}}_{\text{GLV}}}_{\text{Larynx}}^{\text{Dose}}$	-0.010
	${{\text{DVH}}_{\text{V}40}}_{\text{Larynx}}^{\text{Dose}}$	0.081
–	$\text{Intercept}$	0.264

## Appendix D: Study summary and additional prognostic evaluations on weight loss

### [D1] Univariate Cox regression analysis on the nWL difference, pWL difference, and WCS difference

**Table:**

Univariate name	OS			PFS			DMFS		
	p-value	HR	95% CI	p-value	HR	95% CI	p-value	HR	95% CI
nWL difference	0.106	1.15	0.97–1.37	0.099	1.12	1.01–1.25	0.062	1.15	0.99–1.33
pWL difference (× 100)	**0.040**	**1.13**	**1.01–1.27**	**0.016**	**1.09**	**1.02–1.17**	**0.034**	**1.11**	**1.01–1.22**
WCS difference (× 100)	**0.017**	**1.05**	**1.01–1.10**	**0.010**	**1.03**	**1.01–1.06**	**0.025**	**1.04**	**1.01–1.08**

### [D2] Multivariable Cox regression analysis of WCS difference, T stage, and N stage

**Table:**

WCS difference adjusted by T/N stage		p-value	HR	95% CI	C-index
OS	T stage	0.053	2.09	0.99–4.41	78.22%
	N stage	< 0.001	4.51	2.12–9.58	
	WCS difference (× 100)	**0.040**	**1.04**	**1.00–1.09**	
PFS	T stage	0.02	1.66	1.08–2.55	68.64%
	N stage	< 0.001	2.36	1.45–3.83	
	WCS difference (× 100)	**0.037**	**1.03**	**1.00–1.05**	
DMFS	T stage	0.174	1.49	0.84–2.64	68.43%
	N stage	0.025	2.15	1.10–4.21	
	WCS difference (× 100)	**0.049**	**1.04**	**1.00–1.07**	

### [D3] Multivariable Cox regression analysis of pWL difference, T stage, and N stage

**Table:**

Multivariables with pWL difference		*p*-value	HR	95% CI	C-index
OS	T stage	0.048	2.12	1.01–4.48	77.64%
	N stage	< 0.001	4.53	2.13–9.52	
	pWL difference (× 100)	0.079	1.11	0.99–1.24	
PFS	T stage	0.019	1.67	1.09–2.57	68.39%
	N stage	< 0.001	2.35	1.45–3.82	
	pWL difference (× 100)	0.055	1.07	0.99–1.15	
DMFS	T stage	0.17	1.49	0.84–2.65	68.22%
	N stage	0.024	2.16	1.10–4.22	
	pWL difference (× 100)	0.064	1.1	0.99–1.21	
